# Factors Explaining the Severity of Acute Achilles Tendinopathy Among Runners: A Comprehensive Cross-Sectional Analysis

**DOI:** 10.1177/19417381251324929

**Published:** 2025-03-20

**Authors:** Marie-Hélène Lavallée-Bourget, Laurence Roy-Bélanger, María García-Arrabé, Xavier Laurier, Arielle Tougas, Blaise Dubois, Valérie Bélanger, Jean-Sébastien Roy

**Affiliations:** Faculty of Medicine, Université Laval, Quebec City, Quebec, Canada, and Centre Hospitalier Universitaire de Québec - Université Laval, Quebec City, Quebec, Canada; Faculty of Medicine, Université Laval, Quebec City, Quebec, Canada, and Centre Hospitalier Universitaire de Québec - Université Laval, Quebec City, Quebec, Canada; Department of Physiotherapy, Faculty of Medicine, Health and Sports, European University of Madrid, Madrid, Spain; Faculty of Medicine, Université Laval, Quebec City, Quebec, Canada, and Centre Hospitalier Universitaire de Québec - Université Laval, Quebec City, Quebec, Canada; Centre for Interdisciplinary Research in Rehabilitation and Social Integration (Cirris), Quebec City, Quebec, Canada; The Running Clinic, Lac-Beauport, Quebec, Canada; Faculty of Medicine, Université Laval, Quebec City, Quebec, Canada, The Running Clinic, Lac-Beauport, Quebec, Canada, and Centre Hospitalier Universitaire de Québec - Université Laval, Quebec City, Quebec, Canada; Faculty of Medicine, Université Laval, Quebec City, Quebec, Canada, and Centre for Interdisciplinary Research in Rehabilitation and Social Integration (Cirris), Quebec City, Quebec, Canada

**Keywords:** Achilles tendinopathy, diagnostic, psychosocial factors, runners, ultrasound assessment

## Abstract

**Background:**

Achilles tendinopathy (AT) is a prevalent musculoskeletal injury among runners, accounting for approximately 10% of all running-related injuries. AT can result in persistent symptoms and impact the quality of life of runners. The Victorian Institute of Sport Assessment questionnaire (VISA-A) is a widely used self-reported tool for assessing severity of AT. However, the anatomic, biomechanical, psychological, and social factors that influence its score are still poorly understood. The aim of this study is to identify the factors that explain the severity of AT based on the VISA-A score in runners experiencing acute AT.

**Hypothesis:**

The VISA-A score will be explained by both biological and psychosocial factors.

**Study Design:**

Cross-sectional study.

**Level of Evidence:**

Level 4.

**Methods:**

Runners with AT were assessed within 3 months of symptoms onset. The potential explanatory factors assessed included sociodemographic characteristics and medical history, as well as psychosocial, physical, and anatomic (ultrasound imaging) variables.

**Results:**

Participants with AT (n = 84) demonstrated moderate impairments, with a mean VISA-A score of 62.8 (SD, 15.1). Three variables emerged as significant factors explaining AT severity: higher level of kinesiophobia and pain catastrophizing, pain during single-leg jumps, and increased cross-sectional Achilles tendon area on ultrasound imaging. These 3 variables had a moderate capacity (*R*^2^ = 0.47) to explain the VISA-A score.

**Conclusion:**

Pain during single-leg jumps, an increased cross-sectional tendon area assessed by ultrasound, and a high score on kinesiophobia and pain catastrophizing questions are associated with higher VISA scores.

**Clinical Relevance:**

These findings provide the basis for the development of more tailored interventions to improve the quality of life and function of runners with acute AT.

Achilles tendinopathy (AT), whether midportion, preinsertional, insertional, or myotendinous, ranks as one of the most prevalent running-related injuries, accounting for approximately 10% of all such injuries.^19^ Nearly 50% of runners will encounter this injury at some point in their running journey.^21^ The onset of AT is closely related to the repetitive foot strike loading pattern, primarily through running speed and distance. Indeed, the Achilles tendon must withstand forces between 6 and 12.5 times the bodyweight during running. The natural course of AT varies significantly, with approximately one-third of runners still experiencing symptoms beyond 1 year after diagnosis.^22^ Furthermore, between 5% and 37% will cope with persistent symptoms over multiple years, leading to a significantly decline in their quality of life.^4^

Among the various patient-reported outcome measures (PROMs) used to monitor the severity of symptoms and functional limitations in runners with AT, the Victorian Institute of Sport Assessment questionnaire (VISA-A) is the most widely used in both research and clinical practice.^28^ However, PROMs, such as the VISA-A, are subjective tools and reflect the patient’s assessment of their own condition.^30^ As such, clinicians have limited understanding of how sociodemographic data, psychosocial variables, physical examination findings, and ultrasound features impact VISA-A scores. To date, no study has explored the capacity of these factors to explain AT severity based on the VISA-A score.

The clinical diagnosis of AT relies primarily on the report of pain proportional to the applied tensile load, on eliciting a painful response through palpation of the tendon and on reports of very localized pain during weightbearing activities.^3,8,18,24^ In addition to physical examination, ultrasonography has emerged as a reliable diagnostic tool capable of identifying various pathological alterations in the Achilles tendon.^27,35^ However, clinical assessment, particularly the history-taking process, is of paramount importance for obtaining an accurate diagnosis.^33^ In addition, there is an increasing awareness regarding the impact of psychosocial factors on the rehabilitation of tendinopathy.^13^ Addressing the problem using a bio-psycho-social model, avoiding purely biological or mechanical models, provides a more holistic understanding of AT, facilitates informed decision-making for a personalized treatment plan, and is currently seen as the most efficacious approach to chronic pain.^12^

This cross-sectional study aims to identify the sociodemographic characteristics, medical semiology, clinical evaluation, and ultrasound imaging findings that explain symptom severity based on the VISA-A score in runners with acute AT. By adopting an integrated approach encompassing anatomic, biomechanical, psychological, and social factors, we aim to establish a robust foundation for a deeper understanding of the VISA-A score in the running population. This approach may not only enhance our comprehension of the disease but also may facilitate informed clinical decision-making, optimize patient care, and drive positive outcomes.

## Methods

### Participants

This cross-sectional study targeted runners from the Quebec City area who were ≥18 years old, had experienced a recent (<3 months) onset of pain in the Achilles tendon region (midportion, preinsertional, insertional, or myotendinous), had run at least once before the onset of pain, had a VISA-A score of ≤80, and consented to participate in the study. For those reporting recurrent pain, a prerequisite was being pain-free for a minimum of 3 months before the onset of the current symptoms. Participants who had previously undergone surgery or experienced a ruptured Achilles tendon were excluded. This project was approved by the Institutional Review Board of the CIUSSS de la Capitale-Nationale (ref no. 2021-2162), and participants provided written informed consent.

### Study Design

Participants were recruited through a multifaceted approach, which included (1) the institutional email list from Université Laval, (2) advertisements on social media platforms (e.g., Facebook and Instagram), and (3) direct outreach to diverse running communities and sports medicine clinics in Quebec City. All enrolled participants underwent an evaluation at the Centre for Interdisciplinary Research in Rehabilitation and Social Integration (Cirris). This evaluation included a variety of self-reported questionnaires and clinical tests to comprehend the multifaceted nature of AT.

The assessment battery included the following key components:

1. **Sociodemographics and Self-Reported Questionnaires:**
  **Demographic Data:** Sex and age.
  **Standardized Questionnaire on AT History:** Pain localization, time since the onset of the current episode, and training parameters.
  **VISA-A Questionnaire:** The VISA-A is an 8-item questionnaire developed for patients with AT that assesses pain, function, and activity capacities. The total score ranges from 0 to 100, with 0 indicating total incapacity. The VISA-A demonstrates excellent test-retest reliability validity (intraclass correlation coefficient, 0.91).^20^
  **Kinesiophobia and Pain Catastrophizing:** Psychological factors such as kinesiophobia and catastrophizing are associated with increased pain, disability, and poorer outcomes in certain tendinopathies.^23^ To assess these factors, 5 questions were adapted from the National Institutes of Health (NIH) minimum data set for low back pain.^9^ Kinesiophobia was evaluated by asking the participants if they believed that: (1) the evolution of their pain had been favorable, (2) an increase in pain indicates that they should stop physical activity, and (3) it was unsafe for them to be active. Pain catastrophizing was assessed by asking: (1) if they felt their pain was significant and would not improve, and (2) how they foresaw the evolution of their running pain. Four of these questions were rated on a 7-item Likert scale, ranging from “strongly disagree” to “strongly agree.” The last question, regarding pain improvement, was rated on a 5-item Likert scale, ranging from “important deterioration” to “important improvement.” A composite score, derived from the 5 questions, was used in the statistical analyses.
  **Sleep:** Participants provided information about their sleep quality, reported with a 4-item Likert scale, bedtime, wake-up time, and total sleep time.^26^

2. **Clinical Examination:**
  **Anthropometric Data:** Body mass index (BMI) (weight, height).
  ** Heel Raise and Hop Tests:** Participants were asked to perform 3 repetitions of each of the following tests: single-leg heel raise, double-leg jump on the spot, and single-leg jump on the spot. They were asked to rate their pain on an 11-item numerical pain rating scale after each test.
  **Running Analysis:** Running cadence and foot strike angle were analyzed during a 5-minute treadmill run. The characteristics of the footwear were assessed through the calculation of the Minimalist Index.^14^

3. **Ultrasound Evaluation:**

Ultrasound evaluation was performed on the symptomatic AT. Ultrasound images were acquired using a 7.5 to 15 MHz linear array transducer connected to a Logiq 9 ultrasonography system (General Electric). Each participant was positioned prone with their feet hanging off the examination table. The focal zone was adjusted at the level of the Achilles tendon.^7^ Images were obtained in longitudinal and transverse planes with the transducer aligned parallel to the fiber orientation to minimize anisotropy. Tendon thickness and cross-sectional tendon area were measured at the point of maximal tendon thickening. Hyperemia was evaluated by power Doppler.^7^ Videos were recorded with the probe scanning the tendon from its calcaneal insertion to the musculotendinous junction, allowing for a dynamic view and a better understanding of its overall appearance. Images were then reviewed using the method developed by Matthews et al.^25^ Three tendon characteristics (thickness, echogenicity, and vascularity) were analyzed and categorized as normal, reactive, or degenerative. In addition, tendon area was measured through the cross-sectional area (CSA) in square millimeters. The images were also assessed for the presence or absence of paratenon thickening and hyperemia, since paratenon involvement is particularly observed in the acute phase.^29^

### Sample Size

Based on the formula proposed by Green^17^ for determining the minimum number of participants required to perform multiple logistic regression analyses (50 + [8 × number of prognostic factors included in the final model]), and considering that 4 factors would likely be included in the final model, a minimum of 82 participants was needed.

### Statistical Analysis

An initial screening of potential explaining factors was conducted using univariate analysis (independent *t* tests or 1-way analysis of variance [ANOVA]) for nominal and ordinal variables, comparing the VISA-A score for the different categories. For continuous variables, Pearson correlation coefficients were used to assess their correlation with the VISA-A score. Nominal and ordinal variables that exhibited significant differences (*P* < 0.05) in the VISA-A score and continuous variables that showed significant correlation (*P* < 0.05) with the VISA-A were selected as potential explaining factors.

Before entering the potential explaining factors into a logistic regression, the Kaiser-Meyer-Olkin (KMO) index was calculated on the set of variables that included all the potential prognostic factors and the group variable. The KMO index was used to determine the sampling adequacy. When the KMO index is ≥0.6, the logistic regression may be performed with all the potential prognostic factors. When the KMO index is <0.6, some potential prognostic factors must be removed according to the measure of sampling adequacy (MSA) given for each variable.

A first multiple logistic regression was then performed using SAS’s GLMSELECT procedure (selection = stepwise; select = sl; slstay = 0.05; slentry = 0.10) on a model containing only the retained potential explaining factors. However, from a clinical perspective, continuous factors are often impractical, as discrete criteria are better suited to clinical use. For this reason, the continuous variables retained by the second model were dichotomized using the median. Thereafter, a final multiple logistic regression was calculated with the dichotomized version of the set of prognostic factors from the initial model.

## Results

A total of 84 participants with AT were included (23 [27.4%] women and 61 [72.6%] men; mean age, 39 ± 10.4 years; BMI, 25.0 ± 3.4 kg/m^2^). Beginners and experienced runners were included as shown by their running experience (10.8 ± 10.2 years), the number of training sessions per week (3.9 ± 1.5), and the number of kilometers run per week (35.2 ± 27.4) (Table 1); 52% of participants reported a previous episode of AT on the same side. Mean time since the beginning of the current episode was 7.6 weeks (SD, 4.0), and the mean score on the VISA-A was 62.8 (SD, 15.1).

**Table 1.** Participant characteristics (n = 84 runners)

	Mean or n	SD or %
Sociodemographic variables		
Age, y (SD)	39.1	10.4
BMI, kg/m^2^ (SD)	25.0	3.4
Sex, n (%)		
Male	61	72.6
Female	23	27.4
Training habits (SD)		
Running experience, y	10.8	10.2
Distance per week, km	35.2	27.4
Training per week, n	3.9	1.5
Pain		
VISA-A (SD)	62.8	15.1
Previous AT, n (%)	44	52.4
Symptoms duration, weeks (SD)	7.6	4.0
Pain tendon localization, n (%)		
Body	56	66.7
Enthesis	16	19.1
Myotendinous junction	12	14.3

AT, Achilles tendinopathy; BMI, body mass index; VISA-A, Victorian Institute of Sport Assessment questionnaire.

The continuous variables with a significant correlation (*P* < 0.05) with the VISA-A score were pain during single-leg jumps (*r* = −0.59; *P* < 0.001), tendon area (*r* = −0.43; *P* < 0.001), kinesiophobia and pain catastrophizing (*r* = −0.36; *P* < 0.001), BMI (*r* = −0.30; *P* = 0.01), and running experience (*r* = −0.22, *P* = 0.04) (Table 2). The 3 heel raise and jump tests were not only correlated with the VISA-A score but also highly correlated with each other. Therefore, we decided to include into the regression only the test that was most highly correlated with the VISA-A - the single-leg jumps. For the *t* tests and ANOVAs, nominal and ordinal variables that showed significant differences on the VISA-A score (*P* < 0.05) were tendon thickness (*P* = 0.008), pain localization (*P* = 0.02), and echogenicity (*P* = 0.02) (Table 3).

**Table 2.** Association between continuous variable and VISA-A

	Pearson Correlation Coefficients	*P* Value
Pain during single-leg jumps	–0.59	**<0.001**
Tendon CSA	–0.43	**<0.001**
Kinesiophobia and pain catastrophizing	–0.36	**<0.001**
BMI	–0.30	**0.01**
Running experience	–0.22	**0.04**
Age	–0.15	0.18
Number of trainings per week	0.14	0.22
Symptoms duration	0.12	0.29
Cadence	0.11	0.33
Minimalist index	0.09	0.40
Distance per week	0.08	0.49

Bold *P* values indicate significance (*P* < 0.05). BMI, body mass index; CSA, cross-sectional area; VISA-A, Victorian Institute of Sport Assessment questionnaire.

**Table 3.** Mean VISA-A for categorized variables

Variable	Categories	N	VISA-A		*P* Value
			Mean	SD	
Tendon thickness	Degenerative	14	60.4	18.8	**0.008**
	Reactive	33	57.6	14.2	
	Normal	37	68.3	12.6	
Pain tendon localization	Midportion	56	62.4	13.6	**0.02**
	Enthesis	16	56.8	20.1	
	Myotendinous junction	12	72.3	9.4	
Echogenicity	Degenerative	17	58.8	18.6	**0.03**
	Reactive	36	59.7	13.1	
	Normal	31	68.5	13.8	
Vascularity	Degenerative	17	58.3	16.8	0.08
	Reactive	16	58.1	15.4	
	Normal	51	65.8	13.9	
Foot strike	Rearfoot	26	64.0	14.1	0.31
	Forefoot	6	54.0	11.0	
	Middle foot	48	63.6	15.7	
Side of pain	Nondominant	40	64.5	15.1	0.32
	Dominant	44	61.2	15.1	
Quality of sleep	Good	17	60.4	15.8	0.39
	Moderate	46	61.9	16.0	
	Poor	21	66.6	12.0	
Previous Achilles tendon pain	No	40	64.2	14.9	0.41
	Yes	44	61.5	15.3	
Sex	Female	23	62.0	16.6	0.79
	Male	61	63.0	14.6	
Presence of paratenon involvement	No	58	62.8	15.7	0.99
	Yes	26	62.7	13. 8	

Bold *P* values indicate significance (*P* < 0.05). VISA-A, Victorian Institute of Sport Assessment questionnaire.

The univariate tests identified 8 potential explaining factors with a KMO index >0.700. As all MSAs were above 0.700, no potential explaining factors needed to be removed. Eight variables were entered in the first multiple logistic regression: BMI, kinesiophobia/pain catastrophizing, running experience, pain on single-leg jumps, tendon thickness, pain localization, and echogenicity. Three variables came out as significant after the first multiple logistic regression (*P* < 0.05): pain on single-leg jumps (*P* < 0.0001), tendon area (*P* = 0.005), and pain catastrophizing score (*P* = 0.009). These 3 variables had a moderate capacity (*R*^2^ = 0.47) to explain the VISA-A score.

The 3 variables retained by the model were dichotomized using the median. The potential dichotomized explanatory factors for increased AT severity, based on the VISA-A score, were set at a score >2 out of 10 for pain during single-leg jumps, >22 out of 40 for pain catastrophizing, and a tendon CSA of >68 mm^2^. Subsequently, another multiple logistic regression was performed with the dichotomized variables, and the model remained significant, demonstrating a moderate capacity (*R*^2^ = 0.35) to explain the VISA-A score (Tables 4 and 5).

**Table 4.** VISA-A prediction with dichotomized variables

Variables	Degrees of Freedom	Parameter Estimate	Standard Error	*t* value	Pr > |*t*|
Intercept	1	75.75	2.42	31.30	<0.0001
Pain at single-leg jumps (>2 out of 10)	1	–11.28	2.84	–3.97	0.0002
Tendon area (>68 mm^2^)	1	–6.44	2.79	–2.30	0.0238
Kinesiophobia & pain catastrophizing score (>22 out of 40)	1	–7.77	2.80	–2.77	0.0069

VISA-A, Victorian Institute of Sport Assessment questionnaire.

**Table 5.** VISA-A prediction with combinations of dichotomized variables

Pain at Single-leg Jumps	Tendon Area	Kinesiophobia and Pain Catastrophizing Score	VISA-A Predicted Score
≤2	≤68	≤22	75.8
≤2	>68	≤22	69.3
≤2	≤68	>22	68.0
>2	≤68	≤22	64.5
≤2	>68	>22	61.5
>2	>68	≤22	58.0
>2	≤68	>22	56.7
>2	>68	>22	50.3

VISA-A, Victorian Institute of Sport Assessment questionnaire.

## Discussion

The primary objective of this study was to provide a comprehensive insight into the factors explaining the VISA-A score in the acute phase of AT by analyzing a wide range of variables based on 4 crucial pillars: sociodemographic characteristics, self-reported questionnaires, physical examination, and structural assessment using ultrasound imaging. The diversity of the sample in terms of age, sex, running experience, running habits, and pain localization allowed us to conduct a thorough analysis of AT. The results obtained emphasize its multifaceted nature and has implications in how to approach this injury in clinical practice.

The assessment of symptoms and functional limitations, particularly through the VISA-A questionnaire, provides valuable insights into the impact of AT on runners. With a mean VISA-A score of 62.8, participants demonstrated moderate impairments. Our regression analysis identified 3 variables significantly linked to VISA-A score in runners with acute AT. Notably, kinesiophobia and pain catastrophizing emerged as prominent self-reported factors, whereas for physical examination and ultrasound assessment, the presence of pain during single-leg jumps and an increase in Achilles tendon CSA were correlated with VISA-A scores. These variables appear to be pivotal factors in relation to AT severity, highlighting the intricate interplay of various factors in determining AT symptoms and functional impairment.

Significant negative correlations emerged between kinesiophobia/pain catastrophizing scores and VISA-A scores, underscoring the pivotal role of psychological factors in the assessment and treatment of AT. Pain catastrophizing, a maladaptive cognitive-affective response to pain, refers to the inclination to amplify the adverse aspects of a pain experience and envision worst possible outcomes.^23^ Our findings are in alignment with recent research highlighting the profound impact of catastrophizing on pain perception and disability, not only in people with lower limb pathologies^31^ but also in those with upper limb tendon injuries.^11^ This finding underscores the need to address catastrophic thinking in clinical assessment and treatment of AT. Recognizing the impact of kinesiophobia and catastrophizing enables healthcare professionals to implement interventions, such as behavior, mindfulness, or cognitive functional therapies, that address psychological factors, ultimately improving patient outcomes and overall wellbeing.^16,23^

Our study identified a significant correlation between the presence of pain during single-leg jumps and severity of AT. Inclusion of heel raises and hop tests in the evaluation of runners with AT is of paramount importance, as they assess the runner’s ability to withstand monopodal and plyometric loading, which is a crucial component of running. Increased stress on the damaged tendon structure is known to trigger pain, and a hallmark of tendon pain is its intermittent nature, which intensifies with mechanical loading. The presence of obvious pain during activities such as heel raises and jumps can be considered an important indicator of the severity of the problem, highlighting its relevance in clinical diagnostics. Paradoxically, load can induce positive adaptation, and the pathological tendon must adapt to be able to withstand loading.^10^

Increased tendon thickness, assessed through CSA, was identified as a significant structural factor associated to AT severity. Given the superficial location of the AT and the ultrasound accuracy for diagnosing AT, point-of-care ultrasound imaging is an ideal imaging method for evaluating pathological alterations in AT.^1^ A change in tendon sectional area may indicate alterations in tendon integrity or structural composition. In pathological tendons, the increase in bound-water and proteoglycan content can explain the change in thickness or CSA. Furthermore, tendon thickening and increase in extracellular matrix content are an attempt to reduce load on tendon cells.^15^ Our findings are consistent with the study of Stecco et al,^32^ who showed that tendon thickening was correlated positively to AT severity assessed with the VISA-A scale.

Several studies have reported that tendon thickening indicates tendon pathology and can be considered a reliable indicator of tendinopathy.^5,34^ However, pathological changes in the tendon does not always correlate with the presence of pain. This might reflect a structural adaptation to loading, because an increase in CSA decreases the stress on the tendon for the same force.^10^ Otherwise, these morphological changes might be a precursor to the development of AT.

It is important to recognize the limitations of our study. First, the heterogeneity of our patient sample likely introduced variability in responses. Moreover, the percentage of runners using a midfoot or forefoot strike was greater than the usually reported proportions.^2^ This can be attributed to the recruitment of runners, partially from sports medicine clinics; one of the potential interventions involves recommending a shift in the type of foot strike or manipulation of the cadence and impact force, which can result in a modification of foot strike pattern. In addition, relying on self-reported VISA-A questionnaires may not accurately reflect the totality of patient experiences and symptoms, as the psychometric properties of this tool have recently been questioned.^6^

## Conclusion

Pain during single-leg jumps, an increase in tendon CSA assessed by ultrasound, as well as a high score on kinesiophobia and pain catastrophizing questions were linked with higher VISA-A scores. These findings have significant clinical relevance as they provide the basis for the development of more tailored interventions to improve the quality of life and function of runners with acute AT.
